# Vitamin C for COVID-19 Treatment: Have We Got Enough Evidence?

**DOI:** 10.3389/fnut.2022.892561

**Published:** 2022-05-19

**Authors:** Siti Rohaiza Ahmad

**Affiliations:** PAPRSB Institute of Health Sciences, Universiti Brunei Darussalam, Gadong, Brunei

**Keywords:** vitamin C, COVID-19, supplementations, intravenous, treatment

## Introduction

COVID-19, an infectious disease caused by SARS-CoV-2, was discovered at the end of 2019. COVID-19 virus invades the lungs, causing pneumonia. Fever, shortness of breath, and taste loss are common symptoms of COVID-19. About 2–3% of the COVID-19 cases lead to death. Over the past year, scientists and healthcare professionals learned more about the virus. Until today, the battle to find the most suitable treatment strategies for COVID-19 is still ongoing. One of the main areas of debate is nutrition strategies for the cure and prevention of COVID-19.

Vitamin C is a water-soluble nutrient that is essential to humans. Vitamin C intake of about 200 mg per day is recommended to reduce the risk of chronic diseases (1). Consume five servings of fresh vegetables and fruits every day or take a 200 mg vitamin C supplement to acquire 200 mg of vitamin C per day. This recommendation, however, may vary depending on the population.

Since many people believe vitamin C is non-toxic and beneficial to health, it is frequently consumed in large quantities (2). The Tolerable Upper Intake Level (UL) for vitamin C varies according to age group (2). The UL for adults aged 19 and up is 2,000 mg/day of vitamin C. The UL for children aged 1–3 years is 400 mg of vitamin C per day. The UL for those aged 4–6 years is 650 mg/day of vitamin C. The UL for adolescent (14–18 years old) is 1,200 mg/day of vitamin C. However, the UL is not intended to apply to people who are receiving vitamin C under medical supervision.

Acute respiratory distress syndrome, acute cardiac problems, multiple organ dysfunction syndrome, septic shock, and death are possible sequelae in more severe cases of COVID-19 (3). The cytokine storm, in which viral replication produces a massive release of cytokines and other immune-related stimuli, resulting in hyper-inflammation, is thought to cause these problems (4–6). The most common cytokine elevated in critically ill COVID-19 patients was Interleukin-6 (IL-6) (4). A preliminary report suggested that the need for mechanical ventilation is connected to elevated Interleukins 6 (IL-6) levels and that mildly raised IL-6 levels are sufficient to identify COVID-19 individuals at high risk of respiratory failure (7). On the other hand, severe COVID-19 was previously reported to have a larger degree of T-cell proliferation, activation, and cytotoxicity in the late stages of illness (8). As a result, in critically ill COVID-19 patients, suppression of cytokine storms and immune function attacks (hyper-inflammation) may be the appropriate treatment strategy.

Vitamin C is one of the nutrients of interest in COVID-19 cure and prevention. Vitamin C may have a number of immune-modulatory mechanisms that can help to mitigate or reduce COVID-19 pathophysiology (4, 5, 9–11). Vitamin C may act as an antioxidant (9–11). Vitamin C supplementation is thought to help reduce oxidative damage and thus prevent worsening internal vascular endothelial injury (4). It has been proposed that vitamin C also can protect against cytokine attack during SARS-CoV-2 infections (4, 5, 9–11) (Tumor Necrosis Factor). TNF-α and other pro-inflammatory cytokines are reduced by vitamin C, whereas anti-inflammatory cytokines are increased Interleukins 10 (IL-10) (9). In humans, vitamin C treatment reduces IL-6 and inhibits *in vivo* IL-6 release in the endothelium induced by endothelin-1 (ET-1) (4). ET-1 is a potent vasoconstrictor peptide that has also been linked to pneumonia and other lungs diseases. As a result of vitamin C's ability to modulate various immune-modulatory responses, vitamin C was identified as a potential COVID-19 treatment regime. This article aims to report the latest findings on potential applications of vitamin C as a prevention and treatment for COVID-19.

## As Daily Supplements–Prevention Measures

Vitamin C therapy has antiviral effects (9, 12). It is long known that vitamin C is used to combat the common cold (13). According to the famous 1970s Linus Pauling's theory, vitamin C intake help to reduce susceptibility to respiratory tract infections. The theory has remained controversial over the years; however, the sales of vitamin C have increased substantially in the US market (13). A balanced diet can fulfill the daily needs of vitamin C, boosting the immune system and thus reducing susceptibility to respiratory infections (14). However, not much data supported the intake of oral vitamin C supplements to increase immunity (13). Several studies have shown that oral vitamin C supplementations since COVID-19 have increased (15, 16). However, dietary supplements in those studies against COVID-19 were not evidence-based. Most of the respondents were influenced by healthcare professionals, social media, family and friends. Speakman et al. (17) stated that the currently available evidence of vitamin C supplementations' effectiveness was generally small sample sizes and varying doses. Another researcher also corroborated that evidence to justify vitamin C supplements is still lacking and needs further investigation (18).

## As Treatment for Critically Ill Patients

The Vitamin C regime is used to treat critically ill patients with COVID-19. Table 1 shows some recent studies or reports on vitamin C treatment for COVID-19 critically ill patients. An observational and subsequent meta-analysis study among 1,807 patients by Gavrielatou et al. (19) reported that critically ill patients with COVID-19 prescribed vitamin C were not associated with a lower mortality rate. Only six observational studies and five RCTs were included in the meta-analysis. Another latest systematic review and meta-analysis by Ao et al. (20), also reported that short-term intravenous vitamin C treatment did not reduce the risk of severity and mortality in patients with COVID-19. The small number of high-quality studies included in the meta-analysis was one of the limitations highlighted by the researchers. Furthermore, the definition of COVID-19 severity varies between studies. There was also insufficient information about the timing and duration of the therapy in those studies. Similarly, another published work by Kwak et al. (21) showed that there is currently a lack of evidence supporting the therapeutic use of intravenous vitamin C. By which the mortality rate of hospitalized patients was not significantly different between patients treated with intravenous vitamin C and those who did not receive it.

**Table 1:** Shows some of the recent studies or reports on vitamin C treatment for COVID-19 critically ill patients.

**Reference**	**Type of study**	**Total number of participants**	**Principal findings**
Gavrielatou et al. (19)	Observational study and meta-analysis	113 patients (24.8% female). Meta-analysis: six observational and five RCTs, a total of 1,807 patients.	Vitamin C administration was not linked to a lower mortality rate in COVID-19 patients who were critically ill.
Ao et al. (20)	Systematic review and meta-analysis	Seven articles: 3 RCTs and four observational study (sample size range from 32 to 323 patients) Meta-analysis: four observational studies	Compared to the placebo treatment group, vitamin C did not affect disease severity.
Kwak et al. (21)	Systematic review and meta-analysis	Systematic review: 8 articles (four RCTs and four retrospective studies) Studies looking at: outcome on mortality, length of stay at a hospital vs. a control group. Meta-analysis: five studies, in total 186 patients, control groups of 188 patients	There was no statistically significant difference in length of hospital stay and in-hospital mortality rate between critically ill COVID-19 patients and the control group.
Zhang et al. (22)	Pilot trial: randomized, controlled and clinical.	56 patients	No improvement to the invasive mechanical ventilation-free days in 28 days (the number of days patients were extubated following study recruitment).

On the other hand, Shakoor et al. (9) reported that vitamin C supplementations showed positive improvements during COVID-19 infections. On this note, however, no clinical trials looking at the associations of diet and COVID-19 have been reported. On the other hand, some clinical trials are already ongoing to establish the possible link of several nutrients for treatment among COVID-19 patients (9).

In conclusion, due to much-conflicting evidence, this calls for more robust investigations on the impact of vitamin C supplementation therapy on the immune response of patients with COVID-19. Furthermore, many reported studies are pilot stage and lack RCTs (22–25). Hemilla et al. (26), suggested that methodologically sound trials with larger patient populations are required to investigate vitamin C's treatment effects against COVID-19. It was also observed that the duration of intravenous vitamin C treatment varies in the research cited above. Therefore, it is highly debated whether vitamin C poses beneficial therapeutic effects on treating respiratory infections and very sick patients with COVID-19.

## As Treatment-Drug Combinations

Interestingly, several recent studies reported how vitamin C could be used in combinations with other drugs or medications in the COVID-19 treatment regime (Supplementary Table S1–as Supplementary File). A recent report by Sahoo et al. (27) attempted to study the combinations of various anti-HIV viral drugs with vitamin C to combat the COVID-19 virus *via* a computational bioinformatic tool study. The computational investigation showed that the combination of 'darunavir with L-ascorbyl-2,6-dibutyrate or ascorbic acid-2-sulfate' strongly impedes the SARS-CoV-2-main protease as a potential COVID-19 treatment option. This study showed that vitamin C could be potentially used in the COVID-19 treatment regime. However, more trials (*in vivo* or *in vitro* studies) are required to confirm these findings. Another report by Ang et al. (28) showed that the combination of Traditional Chinese medicine with high-dose vitamin C regime showed a faster recovery period, reduced symptoms, improved chest condition and loss of taste improvement. However, only 60 COVID-19 patients were enrolled in this particular study, which thus requires a more robust investigation. Another survey by Tan et al. (29), combined vitamin C administration with another drug, diammonium glycyrrhizinate, prevented the worsening symptoms among COVID-19 patients. In conclusion, to confirm the efficacy of these drugs, however, a large and prospective cohort of patients is required.

## Discussions

Vitamin C is an effective antioxidant with antiviral effects, making it a promising choice for combating SARS-CoV-2 infections. Vitamin C may have a number of immune-modulatory mechanisms that can help to mitigate COVID-19 pathophysiology The applications of vitamin C in terms of COVID-19 cure and prevention include vitamin C supplementations, intravenous vitamin C administration for critically ill patients and the study of combined effects with other antiviral drugs.

The currently available evidence of vitamin C supplementations' effectiveness was generally small sample sizes and varying doses. More studies are required to confirm the effectiveness of dietary vitamin C supplementations against COVID-19.

Apart from that, based on the currently available findings, robust data on the effectiveness of intravenous vitamin C for the treatment of COVID-19 patients is still lacking. The studies condition or criteria also varied greatly. For example, the definition of COVID-19 severity varies between studies. There was also insufficient information about the timing and duration of the therapy in those studies. The vitamin C doses used in the studies also varied. Therefore, studies all over the globe lack standardization and needs to be synchronized. Many of the reported studies on intravenous vitamin C for critically ill patients were found to be at a pilot stage. There is also a lack of RCTs and some are believed to be on the way. Therefore, this calls for more studies to be conducted.

Several recent studies reported how vitamin C could be used in combinations with other drugs or medications in the COVID-19 treatment regime. Many of these studies are still in their early stages and however, pose some promising results. Therefore, in order to confirm the efficacy of these drugs, however, a large and prospective cohort of patients is required.

In conclusion, more research on the effectiveness of vitamin C in COVID-19 prevention and treatment is needed, particularly RCTs and large cohort studies.

## Author Contributions

SA solely wrote the article.

## Conflict of Interest

The author declares that the research was conducted in the absence of any commercial or financial relationships that could be construed as a potential conflict of interest.

## Publisher's Note

All claims expressed in this article are solely those of the authors and do not necessarily represent those of their affiliated organizations, or those of the publisher, the editors and the reviewers. Any product that may be evaluated in this article, or claim that may be made by its manufacturer, is not guaranteed or endorsed by the publisher.
