# Supplementary material for: Vitamin C for COVID-19 Treatment: Have We Got Enough Evidence?
Source: Front Nutr. 2022 May 19;9:892561. doi: 10.3389/fnut.2022.892561 (PMC9161352; doi:10.3389/fnut.2022.892561)
Supplement: Supplementary file 1 [file Table_1.docx]

Table S1: COVID-19 treatment studies using a combination of drugs and vitamin C

| Reference | Type of Study | Drugs tested | Principal Findings |
| --- | --- | --- | --- |
| Sahoo *et al*., 2022 | Computational investigation – using bioinformatics tools | Ten anti-HIV protease inhibitors and vitamin C or L-ascorbic acid. | The combination of 'darunavir with L-ascorbyl-2,6-dibutyrate or ascorbic acid-2-sulfate' strongly impedes the SARS-CoV-2-main protease as a potential COVID-19 treatment option. |
| Ang X *et al*. 2022 | Randomized (Total of 60 patients) | Western Medicine, Traditional Chinese Medicine with vitamin C. | Combined therapy of western medicine, traditional Chinese medicine and high dose of vitamin C – effective outcome on COVID-19 treatment. |
| Tan R *et al*., 2022 | Retrospective observational study (207 patients) | diammonium glycyrrhizinate with vitamin C | A promising candidate for preventing the deterioration of COVID-19 patients, i.e. reduced new-onset complications. |
